# Noninterventional retrospective study of standard-of-care systemic treatment patterns and outcomes in US patients with advanced urothelial carcinoma

**DOI:** 10.1093/oncolo/oyaf071

**Published:** 2025-07-14

**Authors:** Helen H Moon, Melissa Kirker, Anup Abraham, Anna Vlahiotis, Abhijeet Bhanegaonkar, Chiemeka Ike, Darrin Benjumea, Chai Kim, Haiyan Sun, Mairead Kearney, Sanjana Chandrasekar, Benjamin Li, Sheena Thakkar

**Affiliations:** Kaiser Permanente Southern California, Riverside, CA, USA; Pfizer, New York, NY, USA; Genesis Research, LLC, Hoboken, NJ, USA; Pfizer, New York, NY, USA; EMD Serono, Inc., Boston, MA, USAan affiliate of Merck KGaA; EMD Serono, Inc., Boston, MA, USAan affiliate of Merck KGaA; Genesis Research, LLC, Hoboken, NJ, USA; Pfizer, New York, NY, USA; Genesis Research, LLC, Hoboken, NJ, USA; Merck Healthcare KGaA, Darmstadt, Germany; Pfizer, New York, NY, USA; Pfizer, New York, NY, USA; Pfizer, New York, NY, USA

**Keywords:** real-world, locally advanced/metastatic urothelial carcinoma, treatment patterns and outcomes, platinum-based chemotherapy, first-line maintenance avelumab

## Abstract

**Background:**

First-line platinum-based chemotherapy (1L PBC) followed by avelumab 1L maintenance (1LM) in patients without disease progression after 1L PBC is a standard-of-care treatment in locally advanced/metastatic urothelial carcinoma (la/mUC). We examined real-world treatment patterns and outcomes in patients with la/mUC treated in the US and characterized early adoption of avelumab 1LM following US Food and Drug Administration approval in June 2020.

**Materials and methods:**

This retrospective cohort study identified patients ≥ 18 years diagnosed with la/mUC between January 2015 and July 2021 using electronic health records from the Flatiron Health database. Treatment patterns and baseline characteristics were described by type of 1L treatment. Real-world progression-free survival (rwPFS) and real-world overall survival (rwOS) were determined using the Kaplan-Meier method.

**Results:**

A total of 4387 patients were included, with 3706 (84.5%) receiving systemic treatment. The most common 1L therapy was cisplatin-based therapy (33.3%), followed by carboplatin-based (30.9%) and immuno-oncology (IO) therapies (28.0%). Patients treated with 1L cisplatin-based therapy had longer median rwPFS and rwOS from 1L initiation (8.0 and 18.3 months, respectively) vs patients treated with 1L carboplatin-based therapy (6.4 and 13.2 months), or IO therapies (6.1 and 14.2 months). Among eligible patients, early use of avelumab 1LM was 29%. Approximately half (51.7%) of treated patients received second-line (2L) treatment, 16.8% received no 2L treatment, and 31.5% remained on 1L at end of study.

**Conclusion:**

Our findings contribute to our understanding of optimal treatment sequencing options based on individual patient characteristics in a rapidly evolving treatment landscape.

**Implications for practice:**

Despite the growing number of treatment options for patients with locally advanced/metastatic urothelial carcinoma (la/mUC), treatment rates remain low and high attrition persists across several lines of therapy. Real-world evidence on treatment patterns and outcomes with novel first-line (1L) regimens is still maturing, providing an opportunity to identify the most effective 1L treatment and sequencing strategies. Although 1L platinum-based chemotherapy followed by avelumab 1L maintenance is a recommended treatment option, patient characteristics should drive 1L therapy selection. Future real-world studies evaluating treatment patterns and outcomes with longer follow-up are needed to understand the real-world effectiveness of novel 1L therapies.

## Introduction

Bladder cancer, of which urothelial carcinoma (UC) is the most common type, is a substantial contributor to the global cancer burden as the 10th most common cancer and the 13th most deadly.^1,2^ Approximately 11% of patients are diagnosed with advanced disease, defined as locally advanced disease that has spread outside the bladder or metastatic disease that has spread to distant lymph nodes and other sites (la/mUC).^3^

Patients with unresectable la/mUC have especially poor survival outcomes.^4^ In the US, the 5-year estimated survival for patients with distant metastatic muscle-invasive bladder cancer is 7.7% compared with 69.6% and 39.0% for localized and locally advanced disease, respectively.^5^

National Comprehensive Cancer Network (NCCN) guidelines recommend systemic therapy for patients with la/mUC, with choice of therapy dependent on patient characteristics.^6^ Previous real-world studies have shown low usage of systemic therapies, with more than half of patients receiving no systemic therapy.^7^ In addition, utilization of palliative care is low (~4%) in patients with advanced disease.^8^

In recent years, the treatment landscape for la/mUC has changed drastically with the arrival of various immuno-oncology (IO) therapies, fibroblast growth factor receptor (FGFR) inhibitors, and antibody-drug conjugate (ADC) therapies that have been incorporated into treatment guidelines.^6,9^ Beginning in 2017, the first IO therapies for la/mUC were approved in the first-line (1L) setting: the anti–PD-L1 antibody atezolizumab^10^ and the anti–PD-1 antibody pembrolizumab.^11^ Subsequent years saw additional approvals of IO therapies, including avelumab,^12^ durvalumab,^13^ and nivolumab.^14^ Regulatory withdrawal of atezolizumab and durvalumab left the US market with 3 approved IO therapies.^15-17^ In the approximately 20% of patients with la/mUC that have *FGFR* alterations, FGFR inhibitors may be used.^18^ Accelerated approval of the FGFR inhibitor erdafitinib for patients with *FGFR2* or *FGFR3* alterations was granted in 2019, with full approval for *FGFR3* alterations in 2024.^19-21^ In addition, ADCs (small-molecule drugs linked to a target-specific antibody) provide potentially more precise targeting of tumor cells.^22^ Following the voluntary withdrawal of sacituzumab govitecan in October 2024,^23,24^ only one ADC is currently approved for la/mUC by the US Food and Drug Administration (FDA) and recommended by NCCN guidelines: enfortumab vedotin (EV).^6,25^

NCCN-recommended (category 1) treatments in the 1L setting have also undergone significant changes. In 2020, platinum-based chemotherapy (PBC; cisplatin- or carboplatin-based) followed by avelumab 1L maintenance (1LM) in patients without disease progression after PBC was approved by the FDA. This recommendation was based on results from the phase 3 JAVELIN Bladder 100 trial (NCT02603432), which showed significantly prolonged overall survival (OS) and progression-free survival (PFS) with avelumab 1LM.^6,26^ Cisplatin-based therapy is the preferred choice for 1L PBC; carboplatin-based therapy is used as an alternative for the 30%-50% of patients who are ineligible for cisplatin.^27,28^ Ineligibility criteria for cisplatin include Eastern Cooperative Oncology Group performance status (ECOG PS) ≥ 3, creatinine clearance < 30 mL/min, grade ≥ 2 peripheral neuropathy, and New York Heart Association class > 3; or ECOG PS 2 and creatine clearance < 30 mL/min.^29^

More recently, two combination regimens were FDA approved and incorporated into NCCN guidelines: EV plus pembrolizumab (preferred in 2023), and nivolumab plus cisplatin and gemcitabine (recommended in 2024).^6^ EV plus pembrolizumab is now an NCCN preferred regimen (category 1) based on results from the EV-302 trial (NCT04223856), which reported superior outcomes vs cisplatin or carboplatin plus gemcitabine for OS (31.5 vs 16.1 months) and PFS (12.5 vs 6.3 months).^6,30^ In addition, nivolumab plus gemcitabine and cisplatin followed by nivolumab maintenance is now an NCCN-recommended therapy (category 1) based on results from the CheckMate 901 trial (NCT03036098), which reported improved outcomes vs cisplatin plus gemcitabine for OS (21.7 vs 18.9 months) and PFS (7.9 vs 7.6 months).^6,31,32^

Considering the rapidly evolving treatment landscape for la/mUC, it is necessary to characterize the treatment options and outcomes of patients in this setting in a real-world cohort. This study aimed to understand real-world systemic treatment patterns and outcomes in patients with la/mUC, including early adoption of avelumab 1LM since its FDA approval in June 2020.

## Methods

### Data source and study design

This study was a non-interventional, retrospective cohort study of patients with la/mUC in the US using data from the Flatiron Health electronic health record (EHR), a longitudinal, demographically and geographically diverse database containing deidentified patient-level structured and unstructured data from approximately 280 cancer clinics (≈ 800 sites of care).^33-35^ The study period was January 1, 2015, to July 31, 2021 (data cutoff). Patients were identified from January 1, 2015, to April 30, 2021, to reflect the 3-month follow-up requirement. The index date was the la/mUC diagnosis date during the patient identification period (**Supplemental Figure S1**).

### Study population

Patients were categorized into the treated cohort (received systemic treatment) or untreated cohort (did not receive systemic treatment on or after diagnosis within the study period). Patients were ≥ 18 years of age, diagnosed with la/mUC (ICD-9: 188x, 189.1, 189.2, 189.3 or ICD-10: C65x, C66x, C67x, C68.0) with transitional cell pathology, and had ≥ 2 clinical visits documented in the Flatiron Health database on or after January 1, 2015; all patients must have been diagnosed with advanced disease (stage IV) on or after January 1, 2015, or diagnosed with early-stage UC with subsequently development of advanced disease on or after January 1, 2015. Patients in the treated cohort must have received 1L PBC (cisplatin, carboplatin, or oxaliplatin); 1L IO monotherapy or combination therapy (pembrolizumab or atezolizumab); or other 1L systemic treatment including targeted or ADC therapies, as monotherapy or in combination with any drug, and/or they must have received avelumab in 1L, 1LM, second-line (2L), or third- or later-line (3L+) therapy as monotherapy or in combination with any drug within the study period.

Patients were excluded if they had a primary tumor site other than bladder, renal, pelvis, ureter, or urethra; lacked relevant unstructured documents in the Flatiron database; received treatment with any investigational drug during the study period; had > 90 days between dates of la/mUC diagnosis and first structured activity; had < 3 months’ follow-up (ie, last contact < 90 days after index date); and treatment with cisplatin- or carboplatin-based regimens, immuno-oncology, or non-PBC before diagnosis of la/mUC (index date).

### Study measures

Baseline demographics and clinical characteristics were reported by 1L treatment group for the treated cohort and the untreated cohort. Treatment patterns included treatment sequences assessed from 1L to 1LM to 2L based on treatment groups within each line (reported as a categorical variable), and line of therapy (LOT). LOT was based on the predefined definition built into the Flatiron Health database (**Supplemental Table S1**). LOT was validated by the presence of a progression event, which could be indicated by radiographic scan, biopsy, clinical assessment, indication of a mixed response coupled with a change in therapy, or indication of possible pseudoprogression (only in patients receiving IO). In instances where the LOT in the Flatiron Health database did not agree with the validation definition, a key medical expert was consulted, and LOT may have been reassigned. For inclusion in the avelumab 1LM group, patients were required to have received avelumab within 180 days of completing 1L PBC, with no evidence of disease progression between end of 1L PBC and start of avelumab. Evidence of response during 1L was not required. Expert clinical input was used to validate the avelumab 1LM patient-identification algorithm.

Clinical outcomes included real-world overall survival (rwOS), real-world progression-free survival (rwPFS), time to treatment initiation (TTI), time to treatment discontinuation (TTD), and treatment-free interval (TFI; for avelumab 1LM only). RwOS was defined as the time between index date or the start of each LOT to death, as documented in the Flatiron Health database, with censoring at last contact in the database or at study cutoff. RwPFS was defined as the time between index date or the start of each LOT to evidence of first progression or death, with censoring at last contact. TTI was defined as the interval between la/mUC diagnosis date and 1L therapy start date. TTD was defined as the interval between LOT start and end date or censor date, which was in turn defined as the last contact date (of death, visit, administration, or noncancelled order) or end of study, whichever came first. TFI was defined as the interval between discontinuation of 1L and start of 1LM.

### Statistical analysis

Continuous variables were summarized using means, standard deviations, medians, and interquartile ranges. Categorical variables were summarized using frequencies and percentages. Missing data were considered a separate category in all analyses and were described using frequency counts and percentages.

Kaplan-Meier curves were used to estimate rwOS, rwPFS, and TTD. Median rwOS, rwPFS, TTI, and TTD, along with interquartile range or 95% confidence intervals, were reported. Statistical analyses were conducted using SAS version 9.4 (SAS Institute Inc, Cary, NC).^36^

Institutional review board (IRB) approval was covered by the Flatiron Health parent protocol. This study was exempt from additional IRB approval because it was retrospective and noninterventional, using only anonymized data.

## Results

### Baseline demographics and clinical characteristics

**Figure 1.**
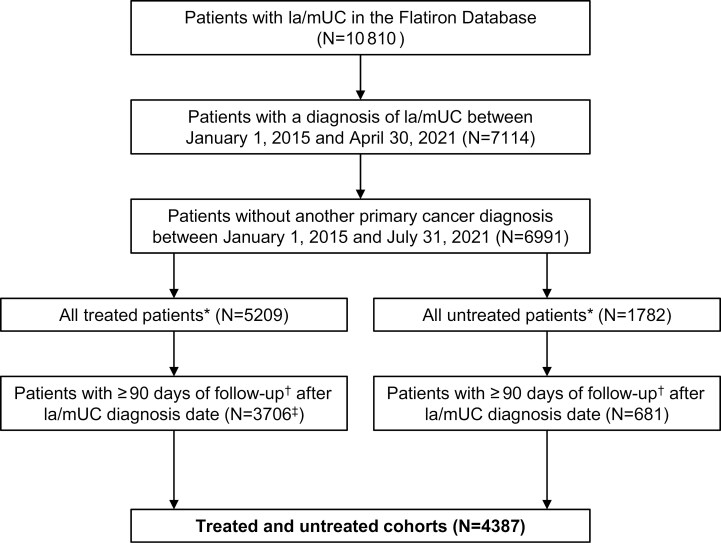
Patient Attrition Flow Chart. **1L**, first line; **la/mUC**, locally advanced/metastatic urothelial carcinoma. *All patients were ≥ 18 years of age. ^†^Unless in the case of death. ^‡^Additional eligibility criteria were met to achieve this number: (1) No systemic treatment for la/mUC in the baseline period (N = 5118), (2) < 180 days between la/mUC diagnosis date and administration of 1L.

Patient selection criteria are described in **Figure 1**. Of 4387 patients identified between January 1, 2015, and April 30, 2021, a total of 3706 (84.5%) received systemic 1L treatment (treated cohort), and 681 (15.5%) did not (untreated cohort) (**Table 1**). Mean age in all patients was 71 years. In both the treated and untreated cohorts, most patients were White (69.8% and 72.1%, respectively) and male (73.4% and 71.1%). Median follow-up time from la/mUC diagnosis was longer for the treated cohort vs the untreated cohort (12.0 vs 10.2 months) (**Supplemental Table S2**). In the treated cohort, patients treated with 1L cisplatin-based therapy had a lower mean age (67.0 years) than patients treated with 1L IO monotherapy (74.6 years); mean age of patients receiving avelumab 1LM was 69.1 years.

**Table 1.** Demographics and Clinical Characteristics of Treated and Untreated Cohorts

Characteristic	All Patients	All Treated Patients	Cisplatin-Based Chemotherapy	Carboplatin-Based Chemotherapy	IO Monotherapy	Other Treatments	Untreated Patients	Avelumab 1LM Patients
	N = 4387	N = 3706	N = 1235	N = 1147	N = 1038	N = 286	N = 681	N = 89
**Age at la/mUC diagnosis, mean (SD), years**	71.0 (8.9)	71.0 (9.0)	67.0 (8.9)	72.1 (8.0)	74.6 (8.2)	71.6 (8.9)	71.0 (8.9)	69.1 (10.1)
**Sex, n (%)**								
Female	1181 (26.9)	984 (26.6)	335 (27.1)	284 (24.8)	290 (27.9)	75 (26.2)	197 (28.9)	20 (22.5)
Male	3205 (73.1)	2721 (73.4)	899 (72.8)	863 (75.2)	748 (72.1)	211 (73.8)	484 (71.1)	69 (77.5)
Unknown	1 (0.0)	1 (0.0)	1 (0.1)	0 (0.0)	0 (0.0)	0 (0.0)	0 (0.0)	0 (0.0)
**Race, n (%)**								
Asian	56 (1.3)	48 (1.3)	22 (1.8)	13 (1.1)	11 (1.1)	2 (0.7)	8 (1.2)	0 (0.0)
Black or African American	199 (4.5)	168 (4.5)	57 (4.6)	53 (4.6)	39 (3.8)	19 (6.6)	31 (4.6)	2 (2.3)
Hispanic or Latino	5 (0.1)	5 (0.1)	1 (0.1)	4 (0.3)	0 (0.0)	0 (0.0)	0 (0.0)	0 (0.0)
White	3076 (70.1)	2585 (69.8)	867 (70.2)	796 (69.4)	716 (69.0)	206 (72.0)	491 (72.1)	54 (60.7)
Other race	677 (15.4)	596 (16.1)	192 (15.5)	183 (16.0)	185 (17.8)	36 (12.6)	81 (11.9)	23 (25.8)
Unknown	374 (8.5)	304 (8.2)	96 (7.8)	98 (8.5)	87 (8.4)	23 (8.0)	70 (10.3)	10 (11.2)
**Region of residence, n (%)**								
Northeast	588 (13.4)	492 (13.3)	172 (13.9)	136 (11.9)	144 (13.9)	40 (14.0)	96 (14.1)	14 (15.7)
Midwest	525 (12.0)	464 (12.5)	157 (12.7)	147 (12.8)	123 (11.8)	37 (12.9)	61 (9.0)	14 (15.7)
South	1969 (44.9)	1723 (46.5)	552 (44.7)	554 (48.3)	489 (47.1)	128 (44.8)	246 (36.1)	41 (46.1)
West	588 (13.4)	511 (13.8)	174 (14.1)	164 (14.3)	144 (13.9)	29 (10.1)	77 (11.3)	13 (14.6)
Other region	46 (1.0)	41 (1.1)	10 (0.8)	13 (1.1)	14 (1.3)	4 (1.4)	5 (0.7)	0 (0.0)
Unknown	671 (15.3)	475 (12.8)	170 (13.8)	133 (11.6)	124 (11.9)	48 (16.8)	196 (28.8)	7 (7.9)
**Site of disease, n (%)**								
Bladder	3377 (77.0)	2825 (76.2)	990 (80.2)	832 (72.5)	794 (76.5)	209 (73.1)	552 (81.1)	73 (82.0)
Renal pelvis	558 (12.7)	485 (13.1)	141 (11.4)	175 (15.3)	127 (12.2)	42 (14.7)	73 (10.7)	9 (10.1)
Ureter	415 (9.5)	366 (9.9)	91 (7.4)	128 (11.2)	116 (11.2)	31 (10.8)	49 (7.2)	7 (7.9)
Urethra	37 (0.8)	30 (0.8)	13 (1.1)	12 (1.0)	1 (0.1)	4 (1.4)	7 (1.0)	0 (0.0)
**Disease grade, n (%)**								
High grade (2/3/4)	3768 (85.9)	3185 (85.9)	1093 (88.5)	951 (82.9)	890 (85.7)	251 (87.8)	583 (85.6)	70 (78.7)
Low grade (1)	207 (4.7)	174 (4.7)	50 (4.0)	58 (5.1)	52 (5.0)	14 (4.9)	33 (4.8)	4 (4.5)
Unknown/not documented	412 (9.4)	347 (9.4)	92 (7.4)	138 (12.0)	96 (9.2)	21 (7.3)	65 (9.5)	15 (16.9)
**Stage at initial diagnosis, n (%)**								
Stage 0	15 (0.3)	13 (0.4)	5 (0.4)	4 (0.3)	4 (0.4)	0 (0.0)	2 (0.3)	1 (1.1)
Stage I	75 (1.7)	66 (1.8)	20 (1.6)	22 (1.9)	20 (1.9)	4 (1.4)	9 (1.3)	3 (3.4)
Stage II	354 (8.1)	286 (7.7)	52 (4.2)	54 (4.7)	149 (14.4)	31 (10.8)	68 (10.0)	6 (6.7)
Stage III	398 (9.1)	335 (9.0)	139 (11.3)	68 (5.9)	103 (9.9)	25 (8.7)	63 (9.3)	3 (3.4)
Stage IV	1618 (36.9)	1415 (38.2)	593 (48.0)	480 (41.8)	246 (23.7)	96 (33.6)	203 (29.8)	40 (44.9)
Unknown/not documented	1927 (43.9)	1591 (42.9)	426 (34.5)	519 (45.2)	516 (49.7)	130 (45.5)	336 (49.3)	36 (40.5)
**Smoking status, n (%)**								
History of smoking	3200 (72.9)	2717 (73.3)	908 (73.5)	850 (74.1)	747 (72.0)	212 (74.1)	483 (70.9)	59 (66.3)
No history of smoking	1165 (26.5)	975 (26.3)	322 (26.1)	292 (25.5)	287 (27.6)	74 (25.9)	190 (27.9)	29 (32.6)
Unknown/not documented	22 (0.5)	14 (0.4)	5 (0.4)	5 (0.4)	4 (0.4)	0 (0.0)	8 (1.2)	1 (1.1)
**PD-L1 status, n (%)**								
Documented								
Negative	370 (8.4)	342 (9.2)	107 (8.7)	115 (10.0)	94 (9.1)	26 (9.1)	28 (4.1)	15 (16.9)
Positive	431 (9.8)	393 (10.6)	117 (9.5)	107 (9.3)	144 (13.9)	25 (8.7)	38 (5.6)	20 (22.5)
Unknown	400 (9.1)	365 (9.8)	106 (8.6)	97 (8.5)	127 (12.2)	35 (12.2)	35 (5.1)	15 (16.9)
Not documented	3186 (72.6)	2606 (70.3)	905 (73.3)	828 (72.2)	673 (64.8)	200 (69.9)	580 (85.2)	39 (43.8)
**GFR (mL/min/1.73m** ^**2**^ **) at la/mUC diagnosis (± 30 days), n (%)**								
< 30	145 (3.3)	128 (3.5)	6 (0.5)	53 (4.6)	57 (5.5)	12 (4.2)	17 (2.5)	0 (0.0)
30-60	945 (21.5)	845 (22.8)	171 (13.8)	319 (27.8)	279 (26.9)	76 (26.6)	100 (14.7)	20 (22.5)
> 60	855 (19.5)	800 (21.6)	363 (29.4)	221 (19.3)	163 (15.7)	53 (18.5)	55 (8.1)	30 (33.7)
Unknown	2442 (55.7)	1933 (52.2)	695 (56.3)	554 (48.3)	539 (51.9)	145 (50.7)	509 (74.7)	39 (43.8)

**1L**, first line; **2L**, second line; **CI**, confidence interval; **GFR**, glomerular filtration rate; **IO**, immune-oncology; **la/mUC**, locally advanced/metastatic urothelial cancer; **OS**, overall survival; **PFS**, progression-free survival.

The most common site of disease in both the treated and untreated cohort was the bladder (76.2% and 81.1%, respectively), and the most common disease stage at initial diagnosis was stage IV (38.2% and 29.8%). In the treated cohort, patients treated with 1L cisplatin- or carboplatin-based therapy had a greater percentage of stage IV disease at initial diagnosis vs patients treated with 1L IO monotherapy (48.0% and 41.8% vs 23.7%, respectively). In both the treated and untreated cohorts, most patients had a history of smoking (73.3% and 70.9%, respectively). Baseline characteristics in patients treated with avelumab 1LM were consistent; the most common site of disease was the bladder (82.0%), the most common disease stage at initial diagnosis was stage IV (44.9%), and most patients had a history of smoking (66.3%).

### Treatment patterns

**Figure 2.**
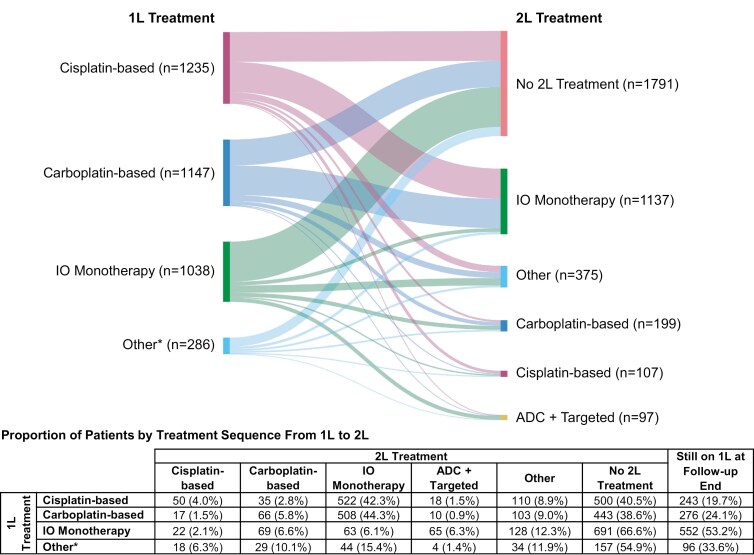
Treatment Patterns in the Treated Cohort. Of patients who received 1L treatment, 4.1% of cisplatin-treated patients and 3.3% of carboplatin-treated patients went on to receive avelumab 1LM treatment; 58.4% of patients who received avelumab 1LM remained on avelumab 1LM at end of follow-up. **1L**, first line; **2L**, second line; **ADC**, antibody-drug conjugate; **IO**, immune-oncology. “Other” includes other PBC (eg, oxaliplatin) and any other treatments that do not fall into other categories. Treatment groups are mutually exclusive. Patients were placed into each group regardless of cross-treatment group combination within this hierarchy: IO, targeted, ADC, cisplatin, carboplatin, any other. Percentages represent row percentages. *Includes ADC + targeted therapy.

In the treated cohort, the most common 1L therapy was cisplatin-based therapy (33.3%), followed by carboplatin-based therapy (30.9%) and IO monotherapy (28.0%) (**Figure 2**); 51.7% of patients (n = 1915) received 2L therapy during the study period, 31.5% (n = 1167) remained on 1L at the end of study, and 16.8% (n = 624) did not receive 2L treatment. In patients treated with 1L PBC, the most common 2L treatment was IO monotherapy, accounting for 42.3% (n = 522) of patients treated with 1L cisplatin-based therapy and 44.3% (n = 508) treated with 1L carboplatin-based therapy. The lowest rates of 2L treatment were observed in patients treated with 1L IO monotherapy, with 66.6% (n = 691) not receiving 2L treatment.

A decrease in the proportion of untreated patients at index was observed over time, from 13.4% in 2015 to 6.1% in 2021 (**Table 2**). The use of PBC decreased by roughly half during this period, from 39.1% in 2015 to 21.9% in 2021. In contrast, the use of IO monotherapy increased from 0.1% in 2015 to 20.6% in 2021.

**Table 2.** Treatment Patterns by Index Year: Treated Cohort and Untreated Cohort

Treatment	2015 (n = 689)		2016 (n = 696)		2017 (n = 707)		2018 (n = 719)		2019 (n = 679)		2020 (n = 706)		2021 (n=191)*	
	n	%	n	%	n	%	n	%	n	%	n	%	n	%
**Untreated**	**163**	**23.7**	**120**	**17.2**	**102**	**14.4**	**109**	**15.2**	**92**	**13.5**	**73**	**10.3**	**22**	**11.5**
**Treated**	**526**	**76.3**	**576**	**82.8**	**605**	**85.6**	**610**	**84.8**	**587**	**86.5**	**633**	**89.7**	**169**	**88.5**
Carboplatin-based chemotherapy	244	46.4	240	41.7	187	30.9	144	23.6	144	24.5	152	24.0	36	21.3
Cisplatin-based chemotherapy	231	43.9	206	35.8	195	32.2	173	28.4	180	30.7	207	32.7	43	25.4
IO monotherapy	1	0.2	76	13.2	189	31.2	255	41.8	220	37.5	223	35.2	74	43.8
Other	50	9.5	54	9.4	34	5.6	38	6.2	43	7.3	51	8.1	16	9.5

**IO**, immune-oncology.

*2021 data had a cutoff date of July 31, 2021.

**Figure 3.**
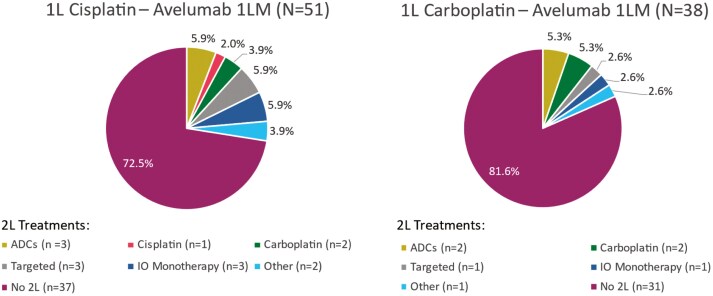
Summary of 2L Treatments Following Avelumab 1LM. **1L**, first line; **1LM**, first-line maintenance; **2L**, second line; **ADC**, antibody-drug conjugate; **IO**, immune-oncology.

A total of 89 patients received avelumab 1LM following treatment with 1L PBC (cisplatin-based therapy, n = 51; carboplatin-based therapy, n = 38). Among these patients, 24% (n = 21) received 2L treatment following avelumab 1LM (cisplatin-based therapy, n = 14; carboplatin-based therapy, n = 7) (**Figure 3**).

### Clinical Outcomes

In the treated cohort, median TTI was 1.3 months in all treated patients and was similar across treatment groups (**Table 3**). Median TTD calculated from 1L initiation was 3.4 months (**Supplemental Figure S2**). Median TTD from initiation of 1L therapy was slightly shorter in patients treated with 1L cisplatin-based therapy vs 1L carboplatin-based therapy (3.1 vs 3.3 months). Median TTD in patients treated with 1L IO monotherapy was 5.0 months. Median TTD from 2L initiation was 2.8 months. Median TTD from initiation of 2L was slightly shorter in patients treated with 2L cisplatin-based vs carboplatin-based therapy (2.4 vs 2.5 months). Median TTD in patients treated with 2L IO monotherapy was 3.1 months. Median TTD from 2L initiation was longest in patients treated with 2L ADCs (3.8 months), followed by 2L targeted therapies (3.2 months). Among the 89 patients who received avelumab 1LM, median TTI was 1.1 months, median TTD was 6.6 months from initiation, and TFI before avelumab was 3.1 weeks (**Table 3**). More than half (58.4%) of patients who received avelumab 1LM remained on avelumab 1LM at end of follow-up.

**Table 3.** TTI, TFI, TTD, rwOS and rwPFS Results: Treated Cohort and Untreated Cohort

Outcome analyses	All Treated Patients	Cisplatin-Based Chemotherapy	Carboplatin-Based Chemotherapy	IO Monotherapy	Antibody-Drug Conjugates (2L only)	Targeted Therapy (2L only)	Other Treatments	Untreated Patients	Avelumab 1LM
**Time to treatment initiation,** **median (IQR), months**	1.3 (1.6)	1.3 (1.4)	1.3 (1.6)	1.3 (1.9)	–	–	1.4 (1.7)	–	1.1 (1.0)
**Treatment-free interval, median (IQR), weeks**	–	–	–	–	–	–	–	–	3.1 (3.3)
**Time to treatment discontinuation,** **median (95% CI), months**									
** From 1L**	3.4 (3.3, 3.5)	3.1 (3.0, 3.3)	3.3 (3.1, 3.4)	5.0 (4.5, 5.6)	–	–	2.5 (2.1, 3.0)	–	3.1 (2.83, 3.53)
** From 1LM**	–	–	–	–	–	–	–	–	6.6 (3.9, 8.9)
** From 2L**	2.8 (2.6, 3.0)	2.4 (1.9, 3.3)	2.5 (1.9, 3.1)	3.1 (2.8, 3.5)	3.8 (2.6, 5.2)	3.2 (1.4, 3.8)	2.1 (2.0, 2.5)	–	6.1 (2.9, 8.0)
**Real-world overall survival (rwOS)**									
**From la/mUC diagnosis**									
Median (95% CI)	16.3 (15.5, 17.1)	20.0 (18.0, 22.0)	14.8 (13.9, 15.8)	15.8 (14.0, 17.2)	–	–	13.1 (11.7, 15.2)	14.8 (13.6, 16.4)	–
6-month rwOS	90.0% (89.1, 91.0)	94.6% (93.3, 95.9)	89.0% (87.2, 90.9)	86.8% (84.7, 88.9)	–	–	86.6% (82.5, 90.7)	87.9% (85.3, 90.4)	97.7% (94.5, 100.0)
12-month rwOS	63.2% (61.6, 64.9)	71.3% (68.5, 74.0)	59.2% (56.2, 62.2)	60.6% (57.4, 63.8)	–	–	55.3% (48.9, 61.6)	58.2% (54.1, 62.3)	87.3% (79.4, 95.2)
24-month rwOS	36.3% (34.5, 38.1)	44.6% (41.4, 47.9)	30.6% (27.5, 33.6)	35.9% (32.4, 39.4)	–	–	26.0% (19.8, 32.2)	35.5% (31.2, 39.7)	72.0% (57.5, 86.5)
**From 1L**									
Median (95% CI)	14.6 (13.9, 15.3)	18.3 (16.4, 19.9)	13.2 (12.2, 14.2)	14.2 (12.4, 15.7)	–	–	11.2 (9.6, 13.9)	–	
6-month rwOS	81.9% (80.6, 83.2)	88.8% (87.0, 90.7)	80.1% (77.7, 82.5)	77.0% (74.4, 79.7)	–	–	77.5% (72.4, 82.6)	–	96.4% (92.4, 100.0)
12-month rwOS	57.3% (55.5, 59.0)	65.5% (62.5, 68.4)	53.4% (50.3, 56.5)	54.8% (51.5, 58.1)			47.3% (40.9, 53.8)	–	84.9% (76.0, 93.8)
24-month rwOS	33.9% (32.1, 35.7)	41.8% (38.5, 45.0)	27.9% (24.9, 30.9)	34.5% (30.9, 38.0)	–	–	24.1% (18.0, 30.3)	–	–
**From 1LM**									
Median (95% CI)	–	–	–	–	–	–	–	–	
6-month rwOS	–	–	–	–	–	–	–	–	86.5% (78.2, 94.9)
12-month rwOS	–	–	–	–	–	–	–	–	68.7% (52.1, 85.4)
**From 2L**									
Median (95% CI)	9.5 (8.9, 10.2)	11.3 (8.9, 19.2)	9.8 (8.9, 11.4)	9.4 (8.4, 10.5)	7.0 (5.7, NE)	6.9 (5.1, 13.5)	9.8 (8.3, 10.9)	–	–
**Real-world progression-free survival (rwPFS)**									
**From la/mUC diagnosis**									
Median (95% CI)	8.5 (8.2, 8.8)	9.5 (9.1, 10.0)	7.7 (7.4, 8.3)	7.1 (6.6, 7.8)	–	–	9.8 (9.1, 11.3)	–	12.7 (10.1, 14.2)
**From 1L**									
Median (95% CI)	7.1 (6.8, 7.4)	8.0 (7.5, 8.4)	6.4 (6.1, 6.8)	6.1 (5.4, 6.8)	–	–	8.4 (7.3, 9.6)	–	10.8 (8.4, 13.1)
**From 1LM**									
Median (95% CI)	–	–	–	–	–	–	–	–	5.6 (3.8, 6.6)
**From 2L**									
Median (95% CI)	3.8 (3.6, 4.1)	7.1 (5.4, 8.7)	5.6 (4.6, 6.4)	3.3 (3.0, 3.5)	4.9 (3.9, 6.8)	3.4 (3.0, 5.2)	4.7 (4.0, 5.4)	–	4.7 (3.1, NE)

**1L**, first line; **1LM**, first-line maintenance; **2L**, second line; **CI**, confidence interval; **IO**, immune-oncology; **la/mUC**, locally advanced/metastatic urothelial cancer; **NE**, not estimable; **rwOS**, real-world overall survival; **rwPFS**, real-world progression-free survival.

Treatment-free interval describes the time between discontinuation of 1L and initiation of 1LM.

**Figure 4.**
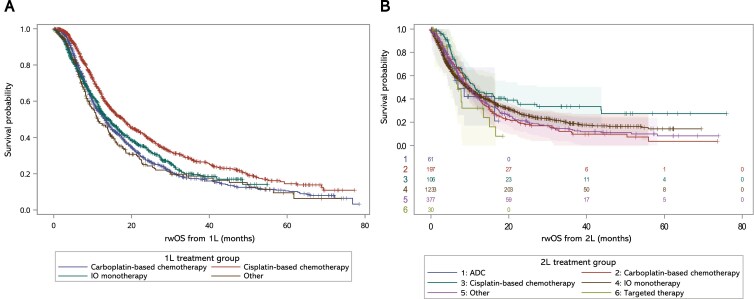
rwOS from (A) 1L Initiation and (B) 2L Initiation in Different Treatment Groups. **1L**, first line; **2L**, second line; **ADC**, antibody-drug conjugate; **IO**, immune oncology; **rwOS**, real-world overall survival.

Patients treated with 1L cisplatin-based therapy had the longest median rwOS from 1L initiation (18.3 months), followed by 1L IO monotherapy (14.2 months) and 1L carboplatin-based therapy (13.2 months) (**Table 3**; **Figure 4**). Patients treated with 2L cisplatin-based therapies had the longest median rwOS from 2L initiation (11.3 months), followed by 2L carboplatin-based therapy (9.8 months) and 2L IO monotherapy (9.4 months). **Supplemental Figure S3** shows rwOS from 1L or 2L initiation in all treated patients. Although median rwOS after avelumab 1LM could not be calculated due to the low number of events, rwOS rates at 6 months and 12 months after 1LM initiation were 86.5% and 68.7%, respectively.

**Figure 5.**
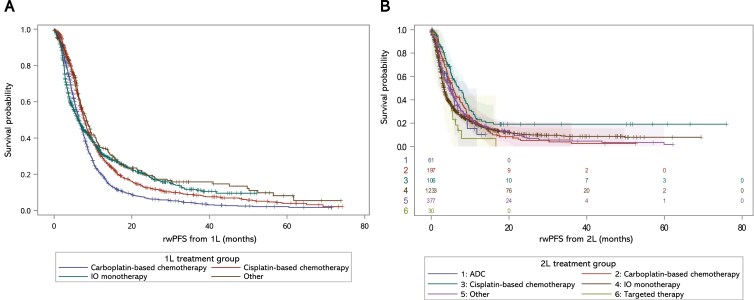
rwPFS From (A) 1L Initiation and (B) 2L Initiation in Different Treatment Groups. **ADC**, antibody-drug conjugate; **1L**, first line; **2L**, second line; **IO**, immune-oncology; **rwPFS**, real-world progression-free survival.

Patients treated with cisplatin-based therapies in 1L had longer median rwPFS from 1L initiation compared with patients treated with carboplatin-based or IO monotherapy (8.0 vs 6.4 and 6.1 months, respectively) (**Table 3**; **Figure 5**). Patients treated with cisplatin-based therapies in 2L had longer median rwPFS from 2L initiation compared with patients treated with carboplatin-based or IO therapies (7.1 vs 5.6 and 3.3 months, respectively). **Supplemental Figure S4** shows rwPFS from 1L or 2L initiation in all treated patients. Median rwPFS from avelumab 1LM initiation was 5.6 months (**Table 3**).

## Discussion

Real-world research in the US has consistently revealed substantial underutilization of systemic treatment in patients with metastatic bladder cancer.^37-40^ Recent real-world studies have found that 25%-50% of patients may not receive systemic 1L therapy.^7,9,41^ Reasons for not receiving systemic treatment include poor physical condition, impaired renal function, or patient preference.^42^ Older age and poor performance status have been statistically correlated with patients not receiving systemic treatment.^40^

However, with the evolving treatment landscape, the rate of 1L systemic treatment is increasing. In a 2022 survey of physicians in the US, 73% believed the proportion of systemic-treated patients has increased with the availability of novel drugs and IO therapies, with physicians estimating that ≥ 75% of existing patients with la/mUC receive systemic therapy.^43^ In agreement, our study found a decrease in the number of untreated patients, from 13.4% in 2015 to 6.1% in 2021. Coupled with the increase in the proportion of treated patients over time was an increase in the proportion of those treated with IO monotherapy over time, accounting for 43.8% of treatments in 2021, although only a modest OS benefit was observed. Compared with other studies,^9,41,44^ the percentage of untreated patients in this study was considerably smaller, likely due to the data source which excludes nationwide coverage and the selection criteria. Median rwOS in the untreated cohort in this study was longer compared with results from a recent analysis that examined Flatiron Health data from 2011 to 2020 (14.8 vs 6.8 months, respectively),^9^ although median follow-up was shorter than in our study (5.3 vs 10.2 months, respectively). Because the study period of our analysis was more recent (2015 to 2021), this finding may suggest that diagnostic or supportive-care advances may have improved survival even in untreated patients.

Since the time of this study, treatment options and recommendations have undergone significant changes including the approval of EV plus pembrolizumab, which is now the preferred 1L regimen for both platinum-eligible and platinum-ineligible patients. However, PBC remains a recommended therapy for la/mUC. Consistent with treatment guidelines at the time of the study, most patients in the treated cohort received PBC (64.2%) and proportions were similar between cisplatin-based and carboplatin-based therapies (33.3% vs 30.9%, respectively), consistent with results from similar real-world studies utilizing the Flatiron Health database, Tempus database, and Medicare FFS claims database.^9,41,45,46^ While usage of 1L IO monotherapy with atezolizumab or pembrolizumab (28%) was similar to rates reported in other real-world studies,^9,41,45^ since the time of the study atezolizumab was withdrawn from the US market, and pembrolizumab monotherapy was restricted to platinum-ineligible patients.^16,47^ Given the rapidly evolving treatment landscape, including the recent approval of EV plus pembrolizumab and nivolumab plus gemcitabine and cisplatin, treatment distributions described in this study likely differ from current treatment distributions and should be assessed in future studies.

Consistent with the platinum eligibility criteria and treatment guidelines at the time of the study, patients treated with 1L cisplatin-based therapy vs 1L carboplatin-based therapy or 1L IO monotherapy were younger (67.0 vs 72.1 and 74.6 years, respectively), more likely to have advanced (stage IV) disease at initial diagnosis (48.0% vs 41.8% and 23.7%), and less likely to have poor kidney function (GFR < 30, 0.5% vs 4.6% and 5.5%; GFR 30-60, 13.8% vs 27.8% and 26.9%). Median rwOS from 1L initiation was longer in patients treated with cisplatin-based therapy vs carboplatin-based therapy or IO monotherapy (18.3 months vs 13.2 or 14.2 months, respectively), as was rwPFS (8.0 months vs 6.4 or 6.3 months), consistent with similar real world studies.^9,41^ In light of the recent guideline updates recommending EV plus pembrolizumab as the preferred therapy for both platinum-eligible and platinum-ineligible patients, the survival outcomes contained in this study provide a relevant benchmark for future real world comparisons.

Approximately half of patients (48.3%) had not received 2L treatment at data cutoff (July 31, 2021), with the majority (65.1%) still on 1L treatment at the end of follow-up. Overall TTD from 1L treatment initiation (3.4 months) and median rwOS from 2L initiation (9.5 months) were equivalent or greater compared with prior real world studies.^41,46,48^ IO monotherapy was the most represented class of 2L therapy (59%) overall, and among those treated with 1L PBC. Most 1L IO monotherapy patients (67%) in our study did not receive 2L therapy, with approximately half (53%) remaining on 1L IO monotherapy at end of follow-up. Since the time of the study, EV monotherapy has subsequently emerged as a preferred 2L treatment but was unavailable during the study period.^6^

The utilization of avelumab 1LM (29%) in this study generally aligns with rates in other real-world studies of 20%,^49^ 29%,^50^ and 37%.^45^ Compared with all treated patients, patients who received avelumab 1LM were younger (71.0 vs 69.1 years, respectively); a lower proportion had high-grade disease (grade 2/3/4) at initial diagnosis (85.9% vs 78.7%) or missing PD-L1 status (70.3% vs 43.8%), and a greater proportion were initially diagnosed at stage IV (38.2% vs 44.9%) or had GFR > 60 (21.6% vs 33.7%). Median rwOS at 12 months with avelumab 1LM was comparable to that reported in Bakaloudi et al (68.7% vs 72.5%)^51^; however, median rwPFS from 1LM initiation was shorter (5.6 vs 9.6 months). The observed difference in median rwPFS could be a result of using different data sources (Flatiron Health vs academic centers in the US and Europe), smaller sample size (89 vs 108 patients), and shorter median follow-up from 1LM initiation (6.0 vs 8.8 months).^51^ In a recent analysis using data from Flatiron Health, median rwPFS and rwOS from avelumab 1LM initiation were 5.1 months and 23.8 months, respectively.^52^ Similar results were reported in other recent real-world studies, including PATRIOT-II,^53^ READY CUP,^54^ and AVENANCE.^55^ In this study more than half (58.4%) of patients who received avelumab 1LM were still receiving it at end of follow-up. In a similar study using the Flatiron Health database, the most common 2L treatment received after avelumab 1LM was EV, accounting for 55% of 2L treatments received, with a median rwPFS and rwOS from 2L EV initiation of 4.9 months and 11.2 months, respectively.^52^ Other recent real-world studies have reported similar findings,^56-58^ supporting EV as a preferred therapy.^6^

In light of the rapidly evolving treatment landscape for la/mUC, this study provides a timely perspective on the treatment patterns and outcomes of patients with la/mUC in the period surrounding avelumab 1LM approval. The strengths of this study include the large, heterogeneous patient population drawn from a curated, reputable database including patients usually ineligible for clinical trials, and builds on existing literature from databases including Flatiron Health,^9,46,49^ among other EHR databases,^45,51^ and findings from clinical trials.^59^ Following the recent approval and recommendation of EV plus pembrolizumab as the preferred 1L therapy for both platinum-eligible and platinum-ineligible patients, the data contained in this study provide a timely source for future comparisons as treatment guidelines evolve and novel treatment options are adopted. While EV plus pembrolizumab is now the preferred 1L therapy, 1L PBC followed by avelumab 1LM remains a recommended treatment sequence for patients when EV plus pembrolizumab is not suitable, or unavailable.^60^ The data contained in this study may provide a useful comparison for future real-world studies including novel therapies, and may help guide optimal treatment sequencing based on patient characteristics.

The results of this study should be interpreted with caution in regards to limitations inherent to the study design including the data source and study period. As with all retrospective database studies, no causal inferences can be made, and findings may not be generalizable to other populations. The lack of patient randomization to treatment may cause confounding. Completeness of data is a fundamental limitation, and misclassification bias may have influenced results, including mortality, which is derived from multiple sources. Patient data may be missing due to clinic visits outside the Flatiron Health network, and patients in certain regions within the US (eg, the Midwest) may be underrepresented. Moreover, data elements may not reflect real-world practice, including differences in categorization of la/mUC by disease stage.

Flatiron Health data are largely derived from community oncology practices; in our study sample, only 12% of patients received care in an academic setting. This finding strongly reflects oncology care in the US, where approximately 85% of patients receive care in a community setting.^61^ Thus, while the distribution of patients in this study between community vs academic centers aligns with real-world observations, results may not be generalizable to patients treated in academic centers outside the US.

Within both the treated and untreated cohorts, information on non-systemic treatments (surgery, radiation, etc.) was not captured, nor was the reasoning behind using or not using certain therapies. In addition, the untreated population (15.5%) may have been underestimated vs populations of other large cohort studies, which reported that approximately half of patients did not receive systemic therapy.^7^ However, a recent survey of US-based oncologists estimated that only 23% of patients had not received systemic therapy.^62^ Thus, with the evolving treatment landscape, the proportion of patients receiving no systemic treatment is likely decreasing, which is reflected in the decreasing proportion of patients who were untreated in this study. Additionally, patients with prior treatment with any platinum-based or IO-based therapies were excluded, and results may not be reflective of patients who develop aUC following treatment for localized disease.

Finally, considering the more recent approval of avelumab 1LM relative to the study period, follow-up for patients treated with avelumab 1LM was limited (median, 6 months), and the study likely only captured a group of early adopters. Future studies with longer follow-up may demonstrate increased utilization of avelumab 1LM and allow assessment of its potential impact on clinical outcomes.

## Conclusion

Historically, there has been a need for novel, efficacious treatments for la/mUC, reflected in low treatment rates, high attrition across treatment lines, and poor clinical outcomes. However, with the rapidly evolving treatment landscape and updates to clinical guidelines, the proportion of patients receiving systemic therapies is increasing. Despite recent treatment guideline updates not captured in this study, including EV plus pembrolizumab as the preferred 1L therapy, PBC followed by avelumab 1LM remains a recommended treatment sequence for patients when EV plus pembrolizumab is not suitable or unavailable.^60^ Future studies with more recent data and longer follow-up are needed to understand optimal treatment sequencing options based on individual patient characteristics.

## Supplementary Material

oyaf071_suppl_Supplementary_Figures_S1_Tables_S1-S2

## Data Availability

The data that support the findings of this study have been originated by Flatiron Health, Inc. Requests for data sharing by license or by permission for the specific purpose of replicating results in this manuscript can be submitted to publicationsdataaccess@flatiron.com.
