# Supplementary material for: Noninterventional retrospective study of standard-of-care systemic treatment patterns and outcomes in US patients with advanced urothelial carcinoma
Source: Oncologist. 2025 Jul 14;30(7):oyaf071. doi: 10.1093/oncolo/oyaf071 (PMC12259535; doi:10.1093/oncolo/oyaf071)
Supplement: oyaf071_suppl_Supplementary_Figures_S1_Tables_S1-S2 [file oyaf071_suppl_supplementary_figures_s1_tables_s1-s2.docx]

**SUPPLEMENTARY MATERIAL**

**Supplemental Table S1: Flatiron LOT rules**

| **LOT** was based on the predefined LOT definition built into the Flatiron Health database. LOT were later validated by the presence of a progression event based on Pfizer’s clinical definition of progression-based LOTs. In instances where the Flatiron Health–based LOT did not agree with Pfizer’s definition of an LOT, a key medical expert was consulted, and LOTs may have been reassigned for those patients.  **Flatiron LOT Rules**  First drug episode of an eligible therapy that is given after or ≤14 days before the index date and after the patient’s start of structured activity.  First eligible drug episode plus other eligible drugs given within 28 days. The name of the regimen for that LOT was the combination of therapies in that line, except for the regimen of methotrexate, vinblastine, doxorubicin, and cisplatin/carboplatin, which was named MVAC.  Looking across all drugs that make up a LOT, when there was a gap of >120 days between any 2 sequential drug episodes, Flatiron rules advanced the LOT number.  For the most recent LOT, the end date was defined as follows:   - For patients with no recorded date of death, the date of last patient-level structured activity (i.e., the last record of patient vitals, medication administrations, or reported laboratory tests/results) - For patients with a recorded date of death, the date of death or of the last patient-level structured activity (i.e., the last record of patient vitals, medication administrations, or reported laboratory tests/results), whichever was later. (Note that for patient privacy and deidentification purposes, date of death was generalized to the last day of the month of death).   When available, the abstracted end date for the oral span was used as the LOT end date instead of an order for an oral therapy.  For all other lines, the end date was defined as the day before the start date of the next LOT.  Exceptions:   - Substitution of cisplatin for carboplatin or vice-versa did not advance the LOT - Substitution of the reference product for a biosimilar or vice versa did not advance the LOT.   Maintenance therapy (not already implemented by Flatiron)   - A regimen was considered maintenance therapy when a patient finished receiving platinum therapy and had no documented progression prior to start of IO monotherapy. |
| --- |

**Abbreviations:** IO, immune-oncology; LOT, line of therapy.

**Supplemental Table S2: Follow-up**

| **Follow-up, months** | **All Patients** | **All Treated Patients** | **Cisplatin-Based Chemotherapy** | **Carboplatin-Based Chemotherapy** | **IO Monotherapy** | **Other Treatments** | **Untreated Patients** | **Avelumab 1LM** |
| --- | --- | --- | --- | --- | --- | --- | --- | --- |
| **From la/mUC diagnosis** |  |  |  |  |  |  |  |  |
| Median (IQR) | 11.8 (14.1) | 12.0 (14.0) | 13.4 (16.4) | 11.9 (13.4) | 11.1 (12.7) | 9.9 (10.7) | 10.2 (14.0) | 11.8 (6.7) |
| **From 1L** |  |  |  |  |  |  |  |  |
| Median (IQR) | 10.4 (14.2) | 10.4 (14.2) | 11.9 (16.8) | 610.1 (13.6) | 9.4 (13.4) | 8.3 (10.5) | – | 9.9 (6.9) |
| **From 1LM** |  |  |  |  |  |  |  |  |
| Median (IQR) | – | – | – | – | – | – | – | 6.0 (5.9) |
| **From 2L** |  |  |  |  |  |  |  |  |
| Median (IQR) | 6.5 (11.5) | 6.5 (11.5) | 6.6 (13.2) | 6.2 (11.6) | 6.6 (8.4) | 6.2 (8.9) | – | 3.6 (4.0) |

**Abbreviations:** 1L, first line; 2L, second line; 1LM, first-line maintenance; CI, confidence interval; IO, immune-oncology; IQR, interquartile range; la/mUC, locally advanced/metastatic urothelial cancer.

**Supplemental Figure S1. Study design**

**
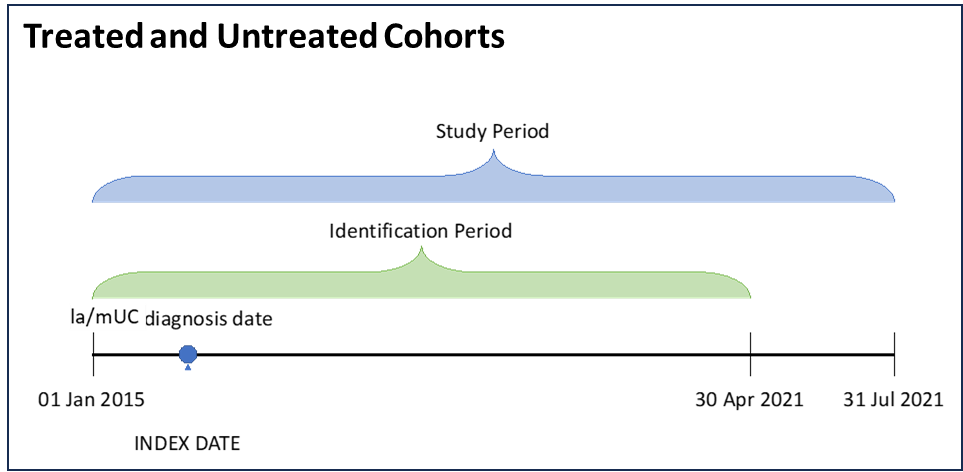
**

**Abbreviation:** la/mUC, locally advanced/metastatic urothelial carcinoma.

**Supplemental Figure S2. Time to treatment discontinuation (A) from 1L initiation in all treated patients; (B) from 1L initiation in 2L treatment groups; (C) from 2L initiation in all treated patients; (D) from 2L initiation in 2L treatment groups.**

**
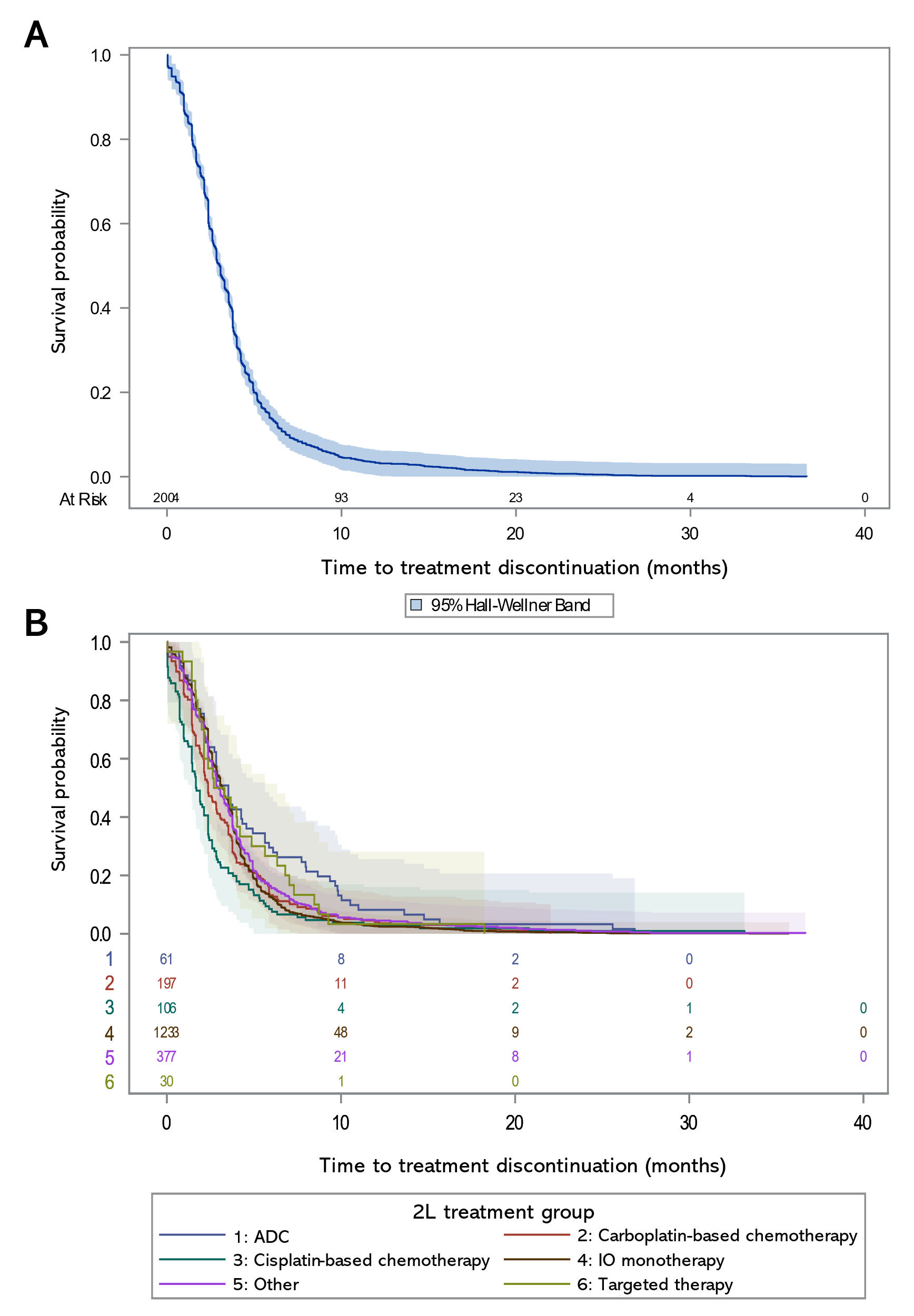
**

**
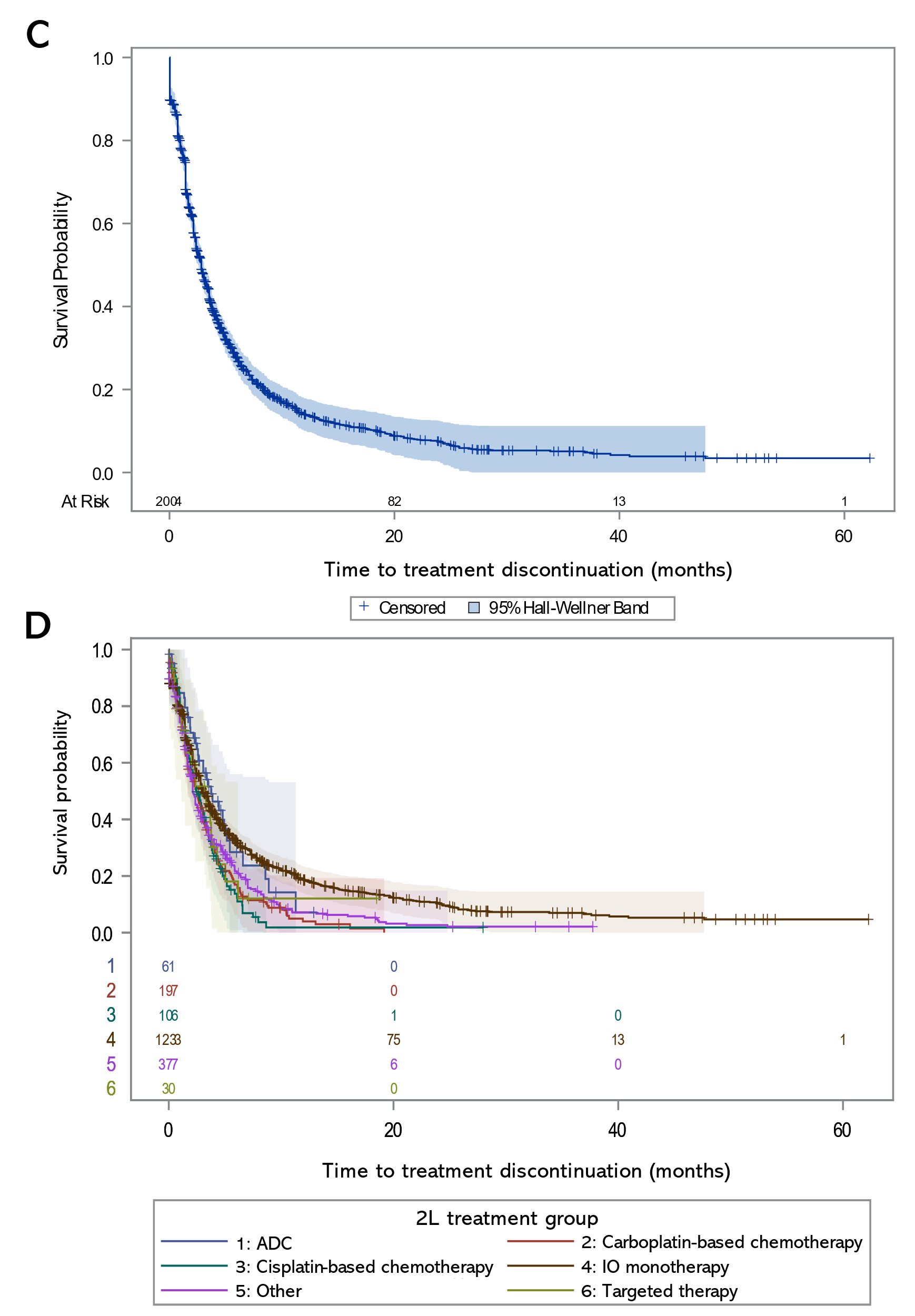
**

**Abbreviations:** 1L, first line; 2L, second line; ADC, antibody-drug conjugate; IO, immune-oncology.

**Supplemental Figure S3. rwOS (A) from 1L initiation and (B) from 2L initiation.**

**
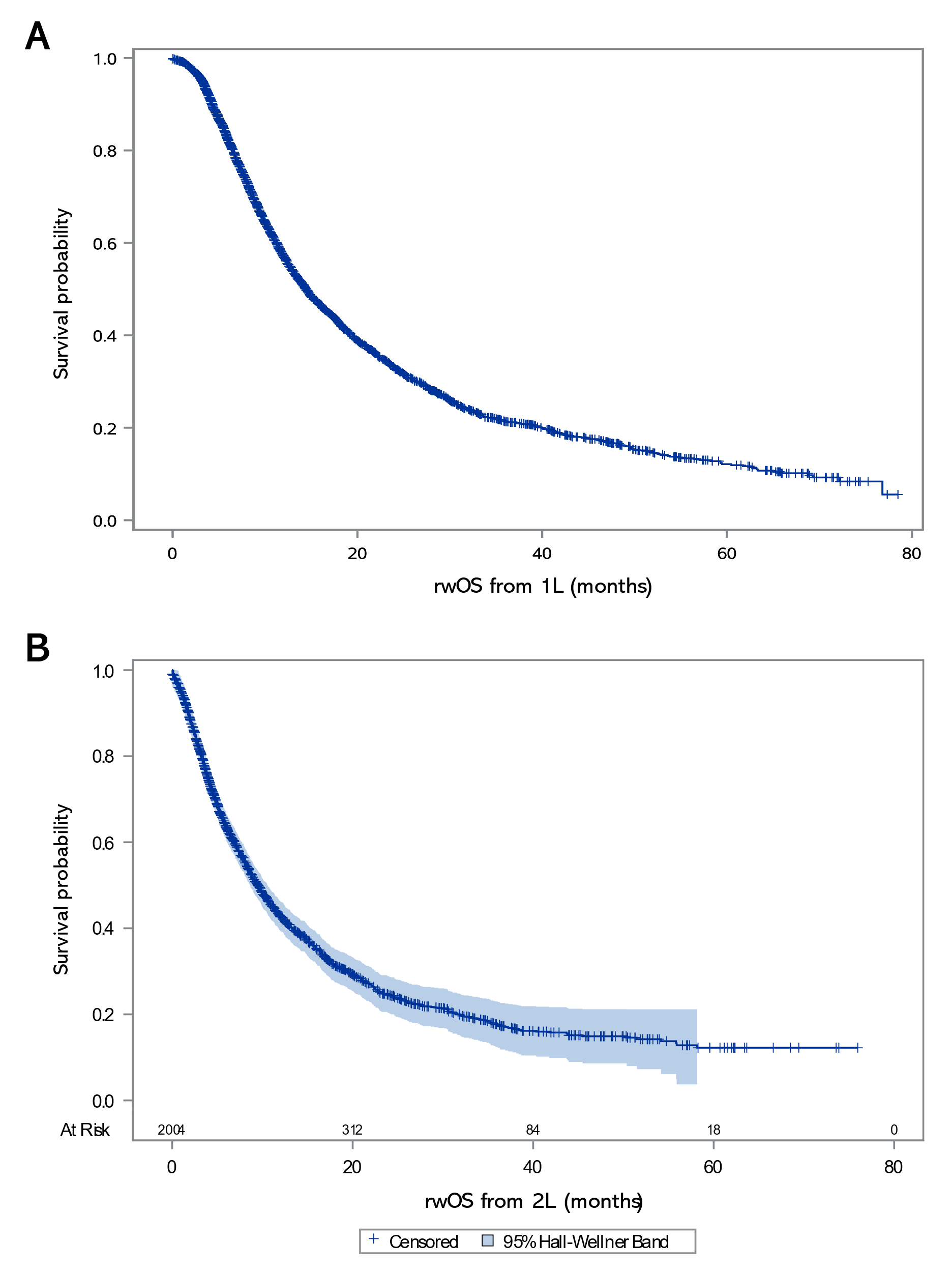
**

**Abbreviations:** 1L, first line; 2L, second line; rwOS, real-world overall survival.

**Supplemental Figure S4. rwPFS (A) from 1L initiation and (B) from 2L initiation.**

**
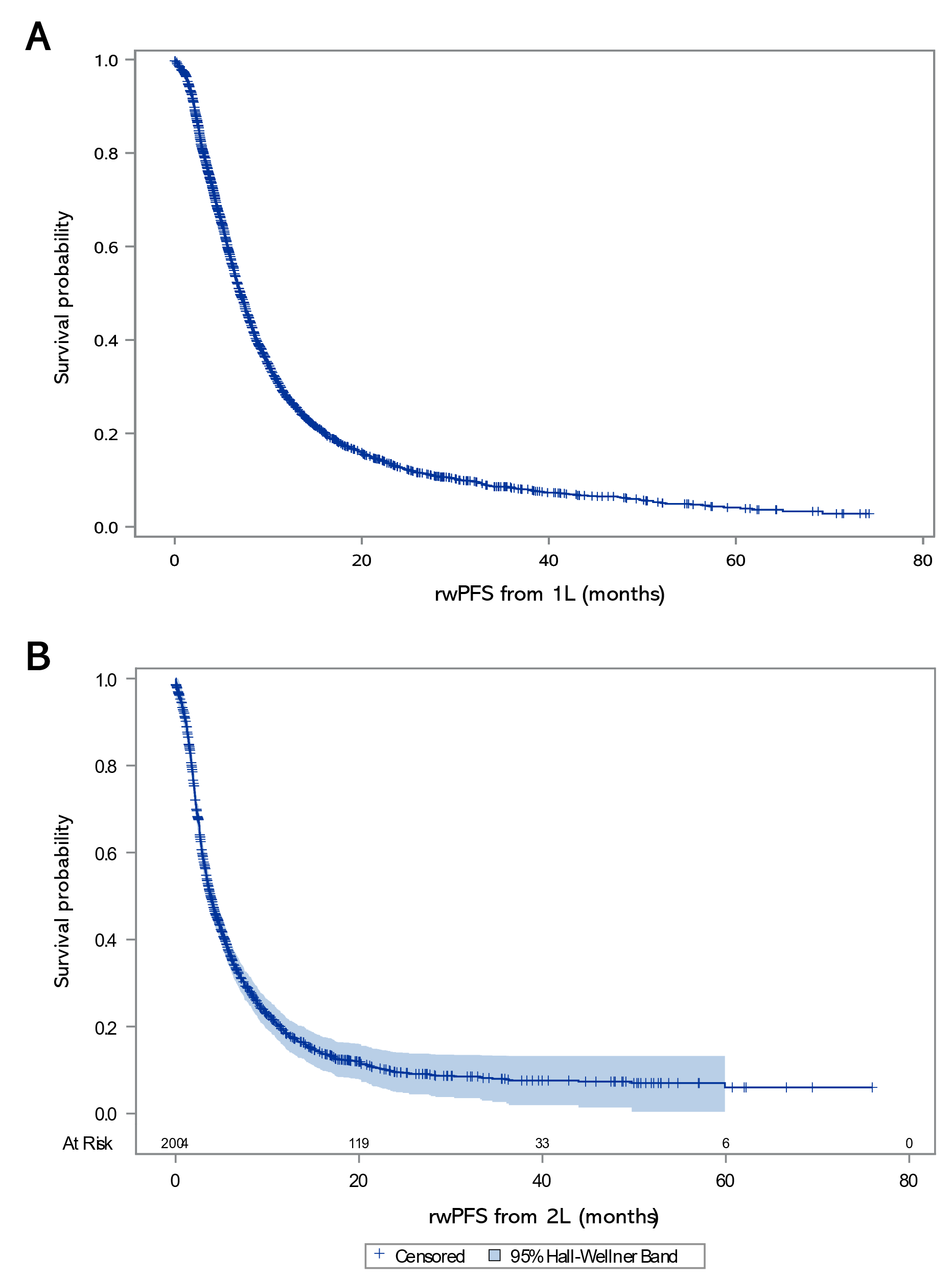
**

**Abbreviations:** 1L, first line; 2L, second line; rwPFS, real-world progression-free survival.
